# German cohort of HCV mono-infected and HCV/HIV co-infected patients reveals relative under-treatment of co-infected patients

**DOI:** 10.1186/1742-6405-11-16

**Published:** 2014-07-01

**Authors:** Claudia Beisel, Martin Heuer, Benjamin Otto, Johannes Jochum, Stefan Schmiedel, Sandra Hertling, Olaf Degen, Stefan Lüth, Jan van Lunzen, Julian Schulze zur Wiesch

**Affiliations:** 1 I. Department of Medicine, Infectious Diseases Unit, University Medical Center Hamburg-Eppendorf, Martinistr. 52, 20246 Hamburg, Germany

**Keywords:** HCV/HIV co-infection, Hepatitis C, PEG interferon/ribavirin treatment

## Abstract

**Background:**

Current German and European HIV guidelines recommend early evaluation of HCV treatment in all HIV/HCV co-infected patients. However, there are still considerable barriers to initiate HCV therapy in everyday clinical practice. This study evaluates baseline characteristics, “intention-to-treat” pattern and outcome of therapy of HCV/HIV co-infected patients in direct comparison to HCV mono-infected patients in a “real-life” setting.

**Methods:**

A large, single-center cohort of 172 unselected HCV patients seen at the Infectious Diseases Unit at the University Medical Center Hamburg-Eppendorf from 2000–2011, 88 of whom HCV/HIV co-infected, was retrospectively analyzed by chart review with special focus on demographic, clinical and virologic aspects as well as treatment outcome.

**Results:**

Antiviral HCV combination therapy with PEG-interferon plus weight-adapted ribavirin was initiated in 88/172 (52%) patients of the entire cohort and in n = 36 (40%) of all HCV/HIV co-infected patients (group A) compared to n = 52 (61%) of the HCV mono-infected group (group B) (p = 0.006). There were no significant differences of the demographics or severity of the liver disease between the two groups with the exception of slightly higher baseline viral loads in group A. A *sustained virologic response* (SVR) was observed in 50% (n = 18) of all treated HIV/HCV co-infected patients versus 52% (n = 27) of all treated HCV mono-infected patients (p = 0.859). Genotype 1 was the most frequent genotype in both groups (group A: n = 37, group B: n = 49) and the SVR rates for these patients were only slightly lower in the group of co-infected patients (group A: n = 33%, group B: 40% p = 0.626). During the course of treatment HCV/HIV co-infected patients received less ribavirin than mono-infected patients.

**Conclusion:**

Overall, treatment was only initiated in half of the patients of the entire cohort and in an even smaller proportion of HCV/HIV co-infected patients despite comparable outcome (SVR) and similar baseline characteristics. In the light of newer treatment options, greater efforts to remove the barriers to treatment that still exist for a great proportion of patients especially with HIV/HCV co-infection have to be undertaken.

## Background

In HIV infected patients, liver-related disease has emerged as a leading cause of morbidity and mortality. The prevalence of HCV/HIV co-infection in the HIV population ranges from 10 to up to 50% [1,2]. On the other hand, HIV co-infection may worsen the course of hepatitis C infection, leading to faster progression of liver fibrosis and cirrhosis, liver failure and development of hepatocellular carcinoma (HCC) [3]. Successful treatment of chronic hepatitis C infection has been shown to stop fibrosis progression, prevent liver-associated diseases and mortality of co-infected patients [4]. Therefore, current German and European HIV guidelines recommend early evaluation of all HIV/HCV co-infected patients for possible HCV treatment. In general, HCV genotype, liver fibrosis, IL-28 haplotypes and quantitative HCV RNA are important baseline predictors of SVR [5]. Demographic factors, histological parameters and treatment related factors can also effect the response to treatment, but in general more than 50% of naïve mono-infected patients will achieve sustained virologic response (SVR) with the current standard therapy (pegylated interferon plus ribavirin) [6,7]. Two large registration trials have shown lower sustained virologic response rates (SVR) for dual therapy in HIV/HCV co-infected patients (~33%) compared to mono-infected patients (47-54%) [8-12]. Higher baseline HCV RNA levels and lower initial ribavirin doses are quoted as possible explanation for the lower SVR seen in co-infected patients [13]. Recent data suggest that higher ribavirin doses are safe and well-tolerated in co-infected patients [14]. Intensified, response guided dual therapy can significantly increase the SVR in co-infected patients [15]. However, less is known about the “real life” treatment outcome of co-infected patients [15].

Great advances have been made with the introduction of the protease inhibitors boceprevir, telaprevir and simeprevir as well as the polymerase inhibitor sofosbuvir that have also been approved for triple therapy in HIV/HCV co-infection [16]. SVR rates of more than 80% have been reported for the triple therapy of naïve patients regardless of the HIV status [16-18].

Despite the unfavorable course of hepatitis C co-infection in HIV positive patients, not all patients are referred to specialists for evaluation for HCV treatment [19]. Furthermore, anti-HCV treatment is only initiated in a subset of all patients for various reasons (e.g. substance abuse, social and psychiatric issues, fear of side effects or drug-drug interactions especially in patients on ART or anticipated low chance of SVR) even in specialized centers [20].

In this “real life” single center study we aimed to summarize the experience and pitfalls of standard dual HCV therapy over the last decade and analyze baseline characteristics, treatment rates and patterns, and outcome of therapy in a German cohort of HCV/HIV co-infected patients. A similar and unselected group of HCV mono-infected patients treated at the same center was evaluated in direct comparison.

## Methods

### Study population and laboratory markers

88 HIV-seropositive and 84 HIV-seronegative, unpaired, unmatched and unselected treatment-naïve patients with chronic HCV infection were enrolled into this mono-center, retrospective cohort study. All patients presented at the Infectious Diseases Outpatient Clinic of the University Medical Center Hamburg-Eppendorf, Germany in the time period from 2000 – 2011 -before directly acting antivirals (DAA’s) became readily available for the treatment of HIV/HCV co-infection. The center is a tertiary care referral center for HIV antiretroviral and HCV antiviral therapy in Germany.

Primary end point of the study was defined as sustained viral response (SVR) by intention-to-treat analysis. Patient charts were analyzed regarding demographics, clinical data (stage of HIV infection; stage of liver fibrosis; therapy regimes and duration; reasons for treatment discontinuation; response to treatment; side effects) as well as laboratory measures such as: HCV genotype; HCV RNA at different time points; HIV RNA; CD4+ T cell count and CD4+/CD8+ ratio at baseline. An assessment of Il28B polymorphisms was not routinely performed during this time period as a standard of care procedure [21]. Hemoglobin levels of both groups were compared at different time points to identify the correlation between treatment response and ribavirin dosage as well as to understand whether co-infected patients have a higher risk of developing anemia under HCV therapy [22].

### Definitions

SVR was defined as an undetectable HCV RNA, 24 weeks after end of treatment. A rapid virologic response (RVR), defined as an undetectable HCV RNA at week 4 of HCV treatment, and early virologic response (EVR), defined as an undetectable HCV-RNA or ≥2 log reduction of HCV-RNA at week 12 of HCV treatment compared to the baseline viral level. A virologic relapse was defined, if HCV RNA decreased and remained below the limit of detection (<50 IU/mL) during treatment but became detectable after end of treatment. Virologic non-response was defined as RNA level HCV RNA level above the limit of detection throughout treatment or a less than 2 log_10_ decline in HCV RNA from baseline until week 12. The stage of HIV infection was defined according to the Center of Disease Control (CDC) – classification, version 1993 [23].

### Statistical analysis

Continuous variables are presented as the mean and standard deviation and categorical parameters are shown as the median and range. The *x*^2^ test and Mann-Withney *U* test were used for analysis of categorical data and of continuous data, respectively. A p value *<0.05* was considered to denote statistical significance. Multiple regression analysis was performed to identify co-variables that might have independently impacted the start of hepatitis C treatment.

## Results

Here, we present treatment results of an unsponsored, single-center study of a German cohort of HIV-negative and HIV-infected patients with chronic HCV infection.

We analyzed a total number of 172 treatment-naïve patients with chronic hepatitis C (genotype 1-4) infection. 88 patients were HIV/HCV co-infected (group A), 84 patients were HCV mono-infected (group B) (Table 1).

**Table 1:** Baseline characteristics of patient cohort

**Characteristics**	**Group A: HCV/HIV co-infected patients**	**Group B: HCV mono-infected patients**	**p-values**
**Patients**, n	88	84	0.666
**Age (y)**, mean (min./max.)	45 (26/64)	45 (20/72)	0.763
**Sex**, male/female (%)	66/22 (75/25)	52/32 (61/38)	0.092
**Genotype**, n (%)			
1	37/88 (42)	49/84 (58)	0.033
2	3/88 (3)	9/84 (10)	0.060
3	25/88 (28)	15/84 (17)	0.102
4	2/88 (2)	5/84 (5)	0.222
Unknwon	21/88 (23)	6/84 (7)	0.003
**Liver biopsy**, n (%)	17/88 (19)	36/84 (42)	0.001
Fibrosis F3 – F4, n (%)	6/17 (35)	10/36 (27)	0.251
**HCV RNA** in IU/ml, mean	4 714 165	3 453 862	0.487
(min./max.)	(3/9 × 10^7^)	(3/3 × 10^7^)	
**HCV RNA > 800 000,** n (%)	66/88 (75)	32/84 (38)	0.001
**HCV RNA < 800 000,** n (%)	22/88 (25)	52/84 (61)	0.001
**Alanine transaminase** in U/l, mean (min./max.)	78 (3/526)	105 (15/610)	0.098
**Start of heptitis C therapy, n (%)**	36 (40)	52 (61)	0.006
**Liver biopsy**, n (%)	10 (27)	27 (51)	0.024
(Fibrosis F3 – F4, n (%)	5 (13)	8 (29)	0.846
**HCV RNA** in IU/ml, mean	4 799 700	3 222 000	0.530
(min./max.)	(800/9 × 10^7^)	(3/2 × 10^7^)	
**HCV RNA > 800 000,** n (%)	12 (33)	21 (40)	0.502
**HCV RNA < 800 000,** n (%)	24 (66)	31 (59)	0.502
**Alanine transaminase** in U/l, mean (min./max.)	94 (29/526)	120 (18/610)	0.321
**Duration of therapy** in weeks, mean (min./max.)	33 (1/152)	36 (10/128)	0.496
Ribavirin (weight adapted) in mg/week, mean	5764,28	7567,2	0.027
Peg-Interferon-alfa 2a, n (%)	27/36 (75)	41/52 (78)	0.672
Peg-Interferon-alfa 2b, n (%)	0/36 (0)	3/52 (5)	0.143
Non-peg-Interferon, n (%)	3/36 (8)	0/52 (0)	0.034

There were no significant differences or relative shifts of the demographics or genotype distributions.

In both groups rates of advanced fibrosis were low (Fibrosis F3 – F4, n (%): 6 (35) versus 10 (27), p = 0.251), however relative fewer liver biopsies were performed in co-infected patients (p < 0.001) (Table 1). While the mean viral load were similar in both groups, more patients with co-infection (n = 66, 75%) had high viral loads (>800 000 IU/ml) than mono-infected patients (n = 32, 38%) (p < 0.001).

**Figure 1:**
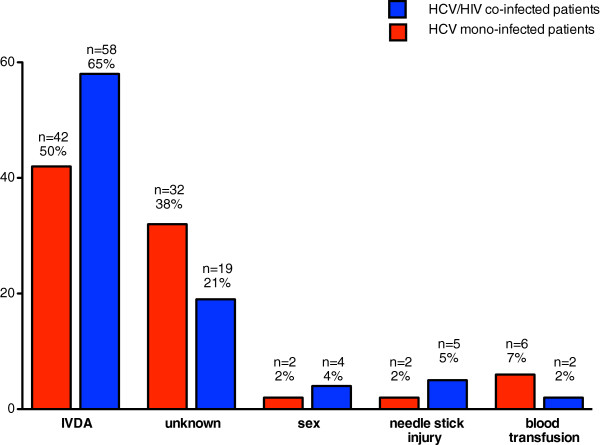
Mode of transmission of hepatitis C Infection.

**Figure 2:**
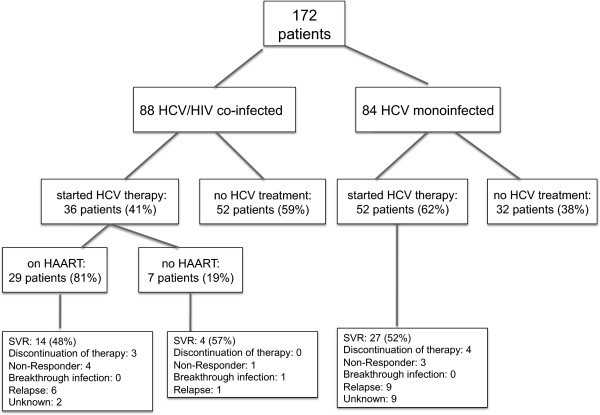
Enrollment, treatment allocation, and follow-up of study participants.

The reported mode of HCV transmission did not differ between groups, with IVDU being the greatest risk factor (Figure 1). The probable mode of HCV transmission was not reported in more patients with mono-infection than co-infection (38% versus 21%) (Table 1).A total of 36 (40%) HCV/HIV co-infected patients started anti-HCV therapy with standard combination treatment regimen with either PEG-IFN alpha 2a or PEG-IFN alpha 2b (weight adapted) for 48 weeks (genotype 1 and 4) or 24 weeks (genotypes 2 and 3) and weight-adapted ribavirin doses of 800-1400 mg, respectively, compared to n = 52 (61%) of mono-infected patients (p = 0.006). Figure 2 summarizes the allocation of treatment and follow-up.

The safety and tolerability of HCV treatment was generally good in both groups and there were no significant differences in discontinuation of therapy. However, only 2/36 (5%) co-infected patients but 9/52 (17%) mono-infected patients were lost to follow-up (p = 0.105).

Overall, SVR rates were comparable in both groups (group A: 18/36 [50%]; group B: 27/52 [52%] (p = 0.859, ns) (Table 2). There were also no differences detected for any of the genotypes (GT) 1 through 4 (GT1: 5/15 [33%] versus 11/27 [40%], GT2 1/2 [50%] versus 7/8 [87%], GT3 9/14 [64%] versus 7/12 [58%], GT4 1/1 [100%] versus 2/4 [50%]). Notably, we neither observed any significant differences of the early virologic response rates, the relapse rates nor differences of the rate of side effects in either group (Table 2). Of note, ART-naïve patients had similar SVR rates compared with those of ART-treated patients (48% versus 57%, respectively). SVR rates were identical for HIV-positive patients with nadir CD4+ T cell counts of 200/μl and above compared to those under 200/μl (Table 2). However, the numbers in these subgroups were too low to conduct a valid statistical analysis.

**Table 2:** Response to HCV therapy

**Response to HCV therapy**	**Group A: HCV/HIV co-infected patients**	**Group B: HCV mono-infected patients**	**p-values**
**SVR all,** n (%)	18/36 (50)	27/52 (52)	0.860
**RVR,** n (%)	9/36 (25)	17/52 (32)	0.440
**EVR,** n (%)	6/36 (17)	7/52 (13)	0.677
SVR GT 1, n (%)	5/15 (33)	11/27 (40)	0.626
SVR GT 2, n (%)	1/2 (50)	7/8 (87)	0.356
SVR GT 3, n (%)	9/14 (64)	7/12 (58)	0.756
SVR GT 4, n (%)	1/1 (100)	2/4 (50)	0.600
GT unknown, n (%)	2/36 (5)	0/52 (0)	0.809
SVR GT 1 + 4, n (%)	6/16 (37)	13/31 (41)	0.770
SVR GT 2 + 3, n (%)	10/16 (62)	14/20 (70)	0.636
**Discontinuation of therapy,** n (%)	3/36 (12)	4/52 (7)	0.305
**Breakthrough infection,** n (%)	1/36 (2)	0/52	0.410
**Non-response,** n (%)	5/36 (13)	3/52 (5)	0.130
**Relapse,** n (%)	7/36 (19)	9/52 (17)	0.482
**Unknown,** n (%)	2/36 (5)	9/52 (17)	0.105
**SVR** HAART treated patients, n (%)	14/29 (48)	n.a.	n.a.
**SVR** non-HAART treated patients, n (%)	4/7 (57)	n.a.	n.a.
**SVR** CD4 Nadir > 200/μl, n (%)	9/18 (50)	n.a.	n.a.
**SVR** CD4 Nadir < 200/μl, n (%)	9/18 (50)	n.a.	n.a.

**Figure 3:**
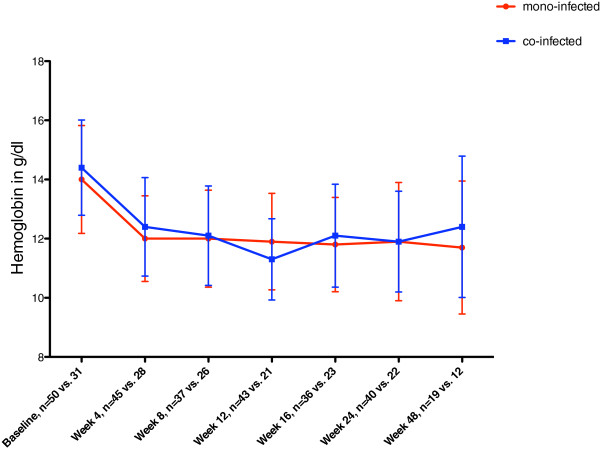
Hemoglobin levels in g/dl of mono-infected (red) versus co-infected (blue) patients during the course of anti-HCV treatment.

We did not find differences in the SVR rates over time in patients treated in the first half in comparison to the second half of the decade (data not shown).The decrease of hemoglobin levels showed no significant differences during the treatment course in both groups (Figure 3). However, group A received a lower cumulated dose of weight-adapted ribavirin than group B (5764,2 mg/week versus 7567,2 mg/week; p = 0,027).

We also compared the demographic and clinical characteristics of the mono and co-infected patients who received treatment or were not treated (Tables 3 and 4). Surprisingly, there were few differences between these groups. Indeed, for the mono-infected patients, there was not a single characteristic significantly different (Table 4).

**Table 3:** Baseline characteristics of HCV/HIV co-infected patients (HCV treated versus untreated)

**Characteristics**	**Group A: HCV/HIV co-infected patients, treated (n = 36)**	**Group B: HCV/HIV co-infected patients, untreated (n = 52)**	**p-values**
**Age**, mean (min./max.)	47 (26/64)	45 (27/61)	0.040
**Sex**, male/female (%)	26/10 (72/28)	40/12 (77/23)	0.617
**Genotype**, n (%)			
1	15 (42)	22 (42)	0.952
2	2 (6)	1 (2)	0.356
3	14 (39)	11 (21)	0.070
4	1 (2)	1 (2)	0.791
Unknown	4 (11)	17 (33)	0.020
**Transmission of HCV**, n (%)			
IVDU	21 (58)	37 (71)	0.212
Transfusion	4 (11)	3 (6)	0.363
Needlestick injury	0 (0)	0 (0)	n.a.
Tatoo	0 (0)	0 (0)	n.a.
Sex	1 (3)	3 (6)	0.508
Unknown	10 (28)	9 (17)	0.241
**CDC Classification**, n (%)			
A1 – A3	13 (36)	10 (19)	0.005
B1 – B3	9 (25)	7 (13)	0.168
C1 – C3	9 (25)	22(42)	0.059
Unknown	5 (13)	14 (26)	0.144
**Liver biopsy, n (%)**	7/36 (19)	5/52 (9)	0.186
Fibrosis F3 – F4, n (%)	5/7 (71)	1/5 (20)	0.029
**HCV RNA** in IU/ml, mean (min./max.)	4800 000 (800/9 × 10^7^)	4 713 000 (3/6 × 10^7^)	0.965
**CD4 Count absolute**, mean	553,1	396,73	0.001
CD4 Count > 200/μl, n (%)	25 (69)	21 (40)	0.007
CD4 Count < 200/μl, n	2 (3,5)	16 (30)	0.004
CD4 Count unknown, n (%)	7 (19)	15 (28)	0.317
**CD4 Count relative**, mean	27,9	22,2	0.001
**CD4 Nadir** in cells/μl, mean	208	194	0.689
**CD4/CD8 Ratio**, mean	0,67	0,49	0.001
**HIV RNA** in copy/ml, mean (minimum/maximum)	9 850 (3/16 × 10^4^)	71 180 (3/12 × 10^5^)	0.015
**HIV Treatment**, n (%)	29 (36)	0 (0)	n.a.
NRTI, n (%)	20/29 (68)	n.a.	n.a.
NNRTI, n (%)	18/29 (62)	n.a.	n.a.
PI, n (%)	12/29 (41)	n.a.	n.a.
Intregrase Inh., n (%)	2/29 (6)	n.a.	n.a.
Entry Inh., n (%)	0 (0)	n.a.	n.a.

**Table 4:** Baseline characteristics of HCV mono-infected patients (HCV treated versus untreated)

**Characteristics**	**Group A: HCV mono-infected patients, treated (n = 52)**	**Group B: HCV mono-infected patients, untreated (n = 32)**	**p-values**
**Age**, mean (min./max.)	44 (22/65)	48 (20/72)	0.112
**Sex**, male/female (%)	34/18 (65/35)	17/15 (53/47)	0.264
**Genotype**, n (%)			
1	27 (52)	22 (69)	0.129
2	8 (15)	1 (3)	0.078
3	12 (23)	3 (9)	0.111
4	4 (8)	1 (3)	0.390
Unknown	1 (2)	5 (16)	0.018
**Transmission**, n (%)			
IVDU	28 (54)	14 (44)	0.369
Transfusion	2 (4)	4 (13)	0.135
Needlestick injury	2 (4)	1 (3)	0.863
Tatoo	2 (4)	0 (0)	0.262
Unknown	18 (35)	13 (41)	0.579
**HCV RNA** in IU/ml, mean (min./max.)	3 222 000 (3/2 × 10^7^)	3 810 000 (3/3 × 10^7^)	0.698
Liver biopsy, n (%)	27/52 (51)	9/32 (28)	0.032
Fibrosis F3 – F4, n (%)	8/27 (29)	2/9 (22)	0.209

In the group of co-infected patients, there were also few differences between the patients who were treated versus treatment naïve patients. In the univariate analysis advanced age (p = 0.04), high CD4+ T cell counts (absolute p < 0.001, relative p = 0.001), a higher mean CD4+/CD8+ ratio (p = 0.001), lower HIV RNA levels (p = 0.015) and thus a CDC classification A1 – A3 (p = 0.005), were associated with the start of HCV treatment in the group of co-infected patients (Table 3). However, multiple regression analysis could not confirm any of those variables as independent predictors for the initiation of HCV treatment in both groups (data not shown).

80% (n = 29) of the co-infected patients receiving anti-HCV treatment were on concurrent HAART. In contrast, none of the HCV untreated patients with HIV/HCV co-infection were on concurrent HAART.

## Discussion

Due to the enhanced risk of rapid progression of liver disease, higher incidence of liver-related complications and higher overall mortality, it is recommended that every HIV/HCV co-infected patient should be evaluated for early HCV treatment [4]. In the present study, we evaluated the treatment decisions and clinical course of HCV/HIV co-infected patients in a real-life treatment setting in a large Northern German university-based cohort in direct comparison to an unselected group of unmatched mono-infected patients treated by the same group of providers.

The first surprising finding is that overall treatment was initiated in only half (88/172) of patients in the entire cohort. Overall SVR was achieved in only 45/88 (51%) of these patients regardless of HIV co-infection status. Our results are in agreement with the results of studies from different parts of the world that show that only a small proportion of the total HCV infected patient population is subjected to treatment and even less are successfully treated [24]. Extrapolating this low number of total successful treatment initiation would have enormous public health consequences [25-27].

This situation might be even worse given the more rapid progression to end stage liver disease in patients co-infected with HCV and HIV [1]. In our current cohort analysis the baseline characteristics of the mono- and co-infected patients with HCV did not differ with regard to stage and severity of liver disease with most patients (group A n = 11; 65% versus group B n = 26; 72%) displaying an early stage of disease (Fibrosis F1- F2). We observed an overall SVR rate of 50% for all genotypes in group A, which is comparable to SVR rates found in randomized clinical trials of peg-INF and ribavirin in HCV/HIV co-infected patients (27% to 40%) [11,12,28]. However, the SVR rates in mono-infected patients (52%) are slightly lower than those reported by a real-life cohort study of mono-infected patients treated by gastroenterologists at the same institution in the same time span (60,9%) [7]. The reasons for these differences are not clear, but we have to keep in mind that the ID clinic is attending for international patients, who might have different IL-28 haplotypes [7]. Also, it has to be noted that 9/84 of the mono-infected patients were lost to follow-up.

The majority of patients received PEG interferon alpha 2a [29], three patients who were treated early in 2000 still received conventional non-pegylated interferon. New direct-acting antiviral agents for hepatitis C genotype 1 infection, such as boceprevir, telaprevir or simeprevir as well as sofosbuvir [17,18,30,31], were not approved yet during the analysed timeperiod.

Despite the satisfactory outcome and an acceptable safety profile, hepatitis C therapy was overall only initiated in half of the patients and even less frequently initiated in HCV/HIV co-infected patients than in HCV mono-infected patients (40% vs. 62%) (p = 0.006) despite the lack of an absolute contraindication according to the chart review in the majority of patients. Reiberger et al. [24] previously also reported a considerable undertreatment of chronic HCV infection in HCV/HIV co-infected patients in a large multicenter study. While our retrospective mono-center study has many limitations that we are aware of, it resembles a “real-life” setting allowing for an evaluation of attitudes towards HCV treatment initiation of the same infectious disease specialists in mono- versus co-infected HCV patients. In our cohort HCV/HIV co-infected patients received less frequently HCV treatment compared to HCV mono-infected patients (p = 0.006) despite being seen by the same specialized ID staff.

Further studies have to look into the reasons of treatment deferral that could range from a potential contraindication to interferon treatment like depression, anemia or other psychiatric illnesses, or possible objections against treatment initiation because of suspected non adherence and fears from the (co-infected) patient’s perspective, as well as relative lower motivation to initiate treatment from the provider perspective because of the fear of inducing severe side effects or the (assumed) lower SVR rates in co-infected patients [32]. Anecdotally, when confronted with the results of the current study the treating ID specialists were surprised by the high SVR rates seen in co-infected patients -especially for the patients that were not on HAART or with lower CD4+ T cell counts. In a follow up study we plan to assess the provider and patient attitudes towards HCV treatment in mono versus co-infected patients by using standardized questionnaires to better understand the barriers to treatment initiation.

Interestingly, in the group of untreated co-infected patients, all patients were HAART naïve. While deferral of HAART treatment might be an indicator for problems of adherence, it is still interesting that only a minority of patients without concurrent HAART regimes received HCV therapy and these patients showed excellent SVR rates. Although guidelines generally recommend selecting patients for HCV treatment whose CD4+ T cell count is high, no study of pegylated interferon plus ribavirin has shown a significant association between CD4+ T cell count less than 350/μl at treatment initiation and SVR to date [33,34]. However, the notion that HCV treatment should only be performed in patients who are on concurrent HAART is a very strong held belief and might be a potential barrier for initiating HCV treatment in HIV co-infection.

Despite comparable rates of development of anemia, HCV/HIV co-infected patients received less ribavirin than mono-infected patients during the course of treatment (5764,3 mg/week versus 7567,2 mg/week; p = 0.027), which may have contributed to the slightly lower SVR rates in co-infected patients (50% versus 52%) [12,15].It seems that treating physicians are more reluctant to use appropriate weight adapted ribavirin dosages in HCV patients co-infected with HIV. However, this treatment pattern was not warranted since baseline hemoglobin levels were comparable in both patient groups (group A: 14,6 g/dl versus group B: 14,5 g/dl) (Figure 3).

In our analysis, HCV treatment was only initiated in half of the patients of the entire cohort at a University based Infectious Diseases Center. Extrapolating these results for Germany, and taking into account the large proportion of patients who statistically do not clear HCV even after one or several courses of therapy is considered, a great number of HIV/HCV co-infected patients are still in need of efficient antiviral treatment even after one decade of dual combination therapy. Altogether greater efforts to understand and potentially to remove the (provider as well as patient) barriers to evaluation and treatment that still exist for a great proportion of patients have to be undertaken [19,27].

Limitations to our study include the relatively small number of patients, its retrospective design and an unequal proportion of men and women in the study population. For similar reasons we also abstained from performing more elaborate statistical tests like multivariate analysis.

The lack of data on IL28B genotypes is another limitation to the study. A recent study at our center in mono-infected patients showed that our center attends only a small number of patients with Asian or African origin and approximately 20% of patients show a CC haplotype, 60% a C/T haplotype and 20% the T/T haplotype (unpublished data). We would assume similar proportions of Il28B haplotypes in our co-infected patients, however cannot exclude the possibility of higher rates of patients with a T/T haplotype as discussed above.

We conclude that overall HCV treatment was only initiated in half of the cohort and even in a smaller proportion of HCV/HIV co-infected patients despite satisfactory outcome and similar baseline characteristics. In the light of newer treatment options [17,18,30,31], greater efforts to understand the barriers to treatment that still exist for a great proportion of patients have to be undertaken especially in patients co-infected with both viruses. Additionally, further efforts have to be undertaken to implement the national and international HIV and HCV guidelines to real life practice. Larger prospective studies are needed to confirm our findings in view of newer treatment option including directly acting antivirals (DAA) for HCV infection.

## Competing interest

The authors declare that they have no competing interests.

## Authors’ contribution

The work presented here was carried out in collaboration between all authors. JvL and JSzW defined the research theme. CB, MH, JSzW, JvL conceived and designed the study. CB, MH, JJ, JSzW and JvL analyzed the data and interpreted the results. CB and JSzW wrote the first draft. JvL gave important input to the manuscript. BO performed the statistical analysis. SS, SH, OD and SL were involved in patient treatment and patient follow-up. All authors read and approved the final manuscript.
